# Variable selection models for genomic selection using whole-genome sequence data and singular value decomposition

**DOI:** 10.1186/s12711-017-0369-3

**Published:** 2017-12-27

**Authors:** Theo H. E. Meuwissen, Ulf G. Indahl, Jørgen Ødegård

**Affiliations:** 1 Norwegian University of Life Sciences, P.O. Box 5003, 1432 Aas, Norway; 2 AquaGen AS, P.O. Box 1240, 7462 Trondheim, Norway

## Abstract

**Background:**

Non-linear Bayesian genomic prediction models such as BayesA/B/C/R involve iteration and mostly Markov chain Monte Carlo (MCMC) algorithms, which are computationally expensive, especially when whole-genome sequence (WGS) data are analyzed. Singular value decomposition (SVD) of the genotype matrix can facilitate genomic prediction in large datasets, and can be used to estimate marker effects and their prediction error variances (PEV) in a computationally efficient manner. Here, we developed, implemented, and evaluated a direct, non-iterative method for the estimation of marker effects for the BayesC genomic prediction model.

**Methods:**

The BayesC model assumes a priori that markers have normally distributed effects with probability $\uppi$ and no effect with probability (1 − $\uppi$). Marker effects and their PEV are estimated by using SVD and the posterior probability of the marker having a non-zero effect is calculated. These posterior probabilities are used to obtain marker-specific effect variances, which are subsequently used to approximate BayesC estimates of marker effects in a linear model. A computer simulation study was conducted to compare alternative genomic prediction methods, where a single reference generation was used to estimate marker effects, which were subsequently used for 10 generations of forward prediction, for which accuracies were evaluated.

**Results:**

SVD-based posterior probabilities of markers having non-zero effects were generally lower than MCMC-based posterior probabilities, but for some regions the opposite occurred, resulting in clear signals for QTL-rich regions. The accuracies of breeding values estimated using SVD- and MCMC-based BayesC analyses were similar across the 10 generations of forward prediction. For an intermediate number of generations (2 to 5) of forward prediction, accuracies obtained with the BayesC model tended to be slightly higher than accuracies obtained using the best linear unbiased prediction of SNP effects (SNP-BLUP model). When reducing marker density from WGS data to 30 K, SNP-BLUP tended to yield the highest accuracies, at least in the short term.

**Conclusions:**

Based on SVD of the genotype matrix, we developed a direct method for the calculation of BayesC estimates of marker effects. Although SVD- and MCMC-based marker effects differed slightly, their prediction accuracies were similar. Assuming that the SVD of the marker genotype matrix is already performed for other reasons (e.g. for SNP-BLUP), computation times for the BayesC predictions were comparable to those of SNP-BLUP.

## Background

Singular value decomposition (SVD) is commonly used in multi-variate statistics to study the explanatory variables and to decompose the design matrix into independent components [1]. In principal component regression (PCR), only the components with the largest singular values are fitted, i.e. PCR considers the smaller components as noise on the explanatory variables, ${\mathbf{X}}$, and thus omits them [1]. This reduction in the number of components is essential in the so-called $k > N$ problems, where the number of explanatory variables ($k$) exceeds the number of records ($N$), because it is not possible to estimate more than $N$ effects from $N$ records, unless random effects are assumed, in which case more than $N$ effects can be predicted.

In genomic selection (GS), matrix ${\mathbf{X}}$ contains the marker genotypes and the number of marker effects ($k$) can greatly exceed the number of phenotypic records, especially in the case of whole-genome sequence (WGS) data. In these cases, the $k > N$ problem is tackled mainly by the use of prior information for the marker effects. For instance, the marker effects can be assumed to have a normal distribution, as in the single nucleotide polymorphism best linear unbiased prediction (SNP-BLUP) model, or they can be assumed to come from a mixture of two distributions with one of them having all its probability density at zero [2]. The latter model assumes with some prior probability $\uppi$ that the marker effect comes from a prior distribution (e.g. the *t*-distribution in BayesB [3] or the normal distribution in BayesC [4]) and with probability (1 − $\uppi$) that the marker effect has no effect on the trait. These models are called variable selection models because they attempt to select the variables that affect the trait [5]. Especially in sequence data, this makes sense biologically, since the causal variates are expected to be contained in the sequence, among many non-causal variates [6]. For these models, straightforward application of PCR does not seem very sensible because all principle components are assumed to be affected by all variates, i.e. PCR does not reduce the number of genotypes involved in the prediction.

Computationally, variable selection models are mostly implemented by using Markov chain Monte Carlo (MCMC) algorithms [2–4], which make them computationally demanding and impractical for large-scale genomic prediction, especially when these involve WGS data. Although some non-MCMC approximations exist, they do not result in quite as accurate predictions as MCMC implementations of these models [7, 8]. Here we show that ${\text{SVD}}$ can simplify the BayesC calculations significantly, make them not MCMC-based, and thus make the analysis of large amounts of WGS data possible in practice. We also compared the results of the ${\text{SVD}}$-based algorithm to those obtained using the MCMC approach. Although the ${\text{SVD}}$ of large amounts of WGS data remains computationally a formidable task, in a companion paper (Ødegård, Indahl, Stranden, Meuwissen: Large-scale genomic prediction using singular value decomposition of the genotype matrix; Accepted in GSE) showed that this task can be performed per chromosome (segment) and thus in parallel.

## Methods

### Applying SVD to the SNP-BLUP model

Generally, we will assume that we are dealing with WGS data. Polymorphisms in the sequence will be called SNPs, although extension to other types of polymorphisms is straightforward, as long as their genotypes can be translated into covariates in a regression model. We will briefly describe the application of ${\text{SVD}}$ to GS, which will also describe our notation. For more details, see [9, 10]. In GS, the general regression model is:

$$ {\mathbf{y}} = {\mathbf{1}}\upmu + {\mathbf{Xb}} + {\mathbf{e,}} $$

where ${\mathbf{y}}$ a $\left( {N \times 1} \right)$ vector of phenotypes; $\upmu$ is the overall mean; ${\mathbf{X}}$ is an $\left( {N \times k} \right)$ matrix of standardized genotypes (see [11]); ${\mathbf{b}}$ is a ($k \times 1$) vector of random SNP effects with $Var\left( {\mathbf{b}} \right) = {\mathbf{I}}\sigma_{b}^{2}$ (the SNP-BLUP model with equal SNP variances is assumed for now); and ${\mathbf{e}}$ is a vector of model residuals ($Var\left( {\mathbf{e}} \right) = {\mathbf{I}}\sigma_{e}^{2}$). Now, the ‘economy’ version of ${\text{SVD}}$ is applied to the matrix ${\mathbf{X}}$ to yield (assuming $k > N$):

$$ {\mathbf{X}} = {\mathbf{USV}}^{{\prime }} , $$

where ${\mathbf{U}}$ is an ($N \times N$) orthonormal matrix of components that describes the family relationships between the animals (${\mathbf{U}}$ contains the eigenvectors of the genomic relationship matrix ${\mathbf{G}}$, with properties: ${\mathbf{U}}^{{\prime }} {\mathbf{U}} = {\mathbf{UU}}^{{\prime }} = {\mathbf{I}}$); ${\mathbf{S}}$ is an ($N \times N$) diagonal matrix of singular values; and ${\mathbf{V}}$ is a ($k \times N$) matrix of components that describe the linkage disequilibrium (LD) structure among the SNPs (properties: ${\mathbf{V}}^{{\prime }} {\mathbf{V}} = {\mathbf{I}}$). Matrix ${\mathbf{V}}$ contains the eigenvectors of the ${\mathbf{X}}^{{\prime }} {\mathbf{X}}$ matrix, which contains the LD between the SNPs as the signed square root of their $r^{2}$ values [1, 12] (“signed” denotes a positive (negative) sign if the correlation between the SNP genotypes is positive (negative)).

Thus, the above regression model can be rewritten as:

$$ \begin{aligned} \varvec{y} & = {\mathbf{1}}\upmu + {\mathbf{USV}}^{{\prime }} {\mathbf{b}} + {\mathbf{e}} \\ & = {\mathbf{1}}\upmu + {\mathbf{USs}} + {\mathbf{e}}, \\ \end{aligned} $$

where ${\mathbf{s}} = {\mathbf{V}}^{{\prime }} {\mathbf{b}}$ and ${\mathbf{b}} = {\mathbf{Vs}}$. In the latter model, ${\mathbf{US}}$ can be seen to represent independent components (i.e. linear combinations) of SNP genotypes, while ${\mathbf{s}}$ represents the effects of these components. At this point, it is possible to omit some of the components with small singular values in ${\mathbf{S}}$, which reflect noise on the estimates of ${\mathbf{X}}$. The variance of the effects of the components is:

$$ Var ({\mathbf{s}} )= Var({\mathbf{V}}^{{\prime }} {\mathbf{b}}) = {\mathbf{V}}^{{\prime }} {\mathbf{V}}\sigma_{b}^{2} = {\mathbf{I}}\sigma_{b}^{2} . $$

When applied to ${\mathbf{s}}$, Henderson’s mixed model equations (MME) [13] become:

1 $$ \left[ {\begin{array}{*{20}c} N & {\mathbf{0}} \\ {\mathbf{0}} & {{\mathbf{S}}^{2} + {\mathbf{I}}\lambda_{b} } \\ \end{array} } \right]\left[ {\begin{array}{*{20}c} {\hat{\mu }} \\ {\hat{s}} \\ \end{array} } \right] = \left[ {\begin{array}{*{20}c} {{\mathbf{1}}^{{\prime }} {\mathbf{y}}} \\ {{\mathbf{SU}}^{{\prime }} {\mathbf{y}}} \\ \end{array} } \right] , $$

where ${\mathbf{S}}^{2} = {\mathbf{SU}}^{{\prime }} {\mathbf{US}}$ (since ${\mathbf{U}}^{{\prime }} {\mathbf{U}} = {\mathbf{I}}$), ${\mathbf{X}}^{{\prime }} {\mathbf{1}} = {\mathbf{0}}$ (a vector of zeros), since the genotypes are standardized, such that the average is 0 for each SNP (i.e. the allele frequencies used are those computed from the data), and $\lambda_{b} = \sigma_{e}^{2} /\sigma_{b}^{2}$. The coefficient matrix of these MME is diagonal, thus computation of the solutions is easy. From these MME, the prediction error variance (PEV) matrix of the components ${\mathbf{s}}$ is: $\left( {{\mathbf{S}}^{2} + {\mathbf{I}}\lambda_{b} } \right)^{ - 1} \sigma_{e}^{2}$, which is a diagonal matrix and thus easy to calculate. From $\hat{s}$, we obtain the estimates of the SNP effects (i.e. as the mean of the posterior distribution):

2 $$ {\hat{\mathbf{b}}} = {\mathbf{V}}\hat{\varvec{s}} . $$

The PEV of the effect of SNP $j$ are readily obtained as:

3 $$ PEV (b_{j} )= {\mathbf{V}}_{j.} ({\mathbf{S}}^{2} + {\mathbf{I}}\lambda_{b} )^{ - 1} {\mathbf{V}}_{j.}^{{\prime }} \sigma_{e}^{2} , $$

where ${\mathbf{V}}_{j}$ is the $j$th row of ${\mathbf{V}}$, which accounts for simultaneous estimation of all SNP effects.

### Application of the BayesC prior

The BayesC prior distribution is a mixture distribution [4]:

$$\text{with}\,\text{prior}\,\text{probability}\, \uppi{:}\;{b_{j}}\sim\,N(0,\sigma^{2})$$

$$\text{and}\,\text{with}\,\text{prior}\,\text{probability}\, (1-\uppi) : b_{j} = 0,$$

where $\sigma^{2}$ is approximately the largest variance a SNP effect is expected to have. E.g., the largest SNP effects are expected to have a variance of ~ 0.001* $\sigma_{g}^{2}$, where $\sigma_{g}^{2}$ is the additive genetic variance. Suitable values for $\uppi$ and/or $\sigma^{2}$ can be obtained by cross-validation (for appropriate cross-validation schemes see [14]).

Consider estimation of the effect of SNP $j$, $b_{j}$. The model is:

$$ \varvec{y} = {\mathbf{1}}\upmu + b_{j} {\mathbf{x}}_{j} + {\boldsymbol{ \in },} $$

where ${\mathbf{x}}_{j}$ is the $j$th column of the genotype matrix ${\mathbf{X}}$ and ${\mathbf{ \in }}$ is a vector of residuals, which includes the effects of all other SNPs and the environmental effects, ${\mathbf{e}}$. Thus, $Var ({\mathbf{ \in }} )= {\mathbf{I}}\sigma_{e}^{2} + {\mathbf{G}}_{ - j} = {\mathbf{R}}\sigma_{e}^{2}$, where ${\mathbf{G}}_{ - j}$ is the genetic variance times the genomic relationship matrix based on all SNPs except SNP $j$, and with ${\mathbf{R}} = {\mathbf{I}} + {\mathbf{G}}_{ - j} /\sigma_{e}^{2}$. Strictly, ${\mathbf{R}}$ depends on SNP $j$ but since the effect of a single SNP on the overall ${\mathbf{G}}$ matrix is expected to be small, we will assume that ${\mathbf{R}}$ is approximately independent of $j$. Then, the MME for the estimation of the effect of SNP $j$ is:

4 $$ \left( {{\mathbf{x}}_{j}^{\varvec{'}} {\mathbf{R}}^{ - 1} {\mathbf{x}}_{j} + \lambda } \right)\hat{b}_{j} = {\mathbf{x}}_{j}^{{\prime }} {\mathbf{R}}^{ - 1} ({\mathbf{y}} - {\mathbf{1}}\upmu) , $$

where ${\mathbf{x}}_{j}$ is the $j$th column of genotype matrix ${\mathbf{X}}$, and the variance ratio $\lambda = \sigma_{e}^{2} /\sigma^{2}$. Note that these MME are the same as for the SNP-BLUP model, except for the variance ratio, which is $\lambda_{b} = \sigma_{e}^{2} /\sigma_{b}^{2}$ for the latter. The PEV of the estimate of the effect of SNP $j$ is:

5 $$ PEV (b_{j} )= \left( {{\mathbf{x}}_{j}^{\varvec{'}} {\mathbf{R}}^{ - 1} {\mathbf{x}}_{j} + \lambda } \right)^{ - 1} \sigma_{e}^{2} . $$

Again, this is the same as for the SNP-BLUP model, except that the variance ratio is $\lambda_{b}$ instead of $\lambda$. For the SNP-BLUP model, we can calculate $PEV\left( {b_{j} } \right)$ using Eq. (3). And, since we assume that $\lambda_{b}$ and $\sigma_{e}^{2}$ are known, we can solve for the ${\mathbf{x}}_{j}^{{\prime }} {\mathbf{R}}^{ - 1} {\mathbf{x}}_{j}$ term in Eq. (5), which represents the effective number of records that contribute to the effect estimate for SNP $j$. By combining ${\mathbf{x}}_{j}^{{\prime }} {\mathbf{R}}^{ - 1} {\mathbf{x}}_{j}$, the SNP-BLUP estimate $\hat{b}_{j}$ [from Eq. (2)], and the variance ratio $\lambda$, we can compute the right-hand-side of Eq. (4): $\mathbf{x}_{j}^{\prime } \mathbf{R}^{ - 1} \left( \mathbf{y} - \mathbf{1}\upmu \right)$, which is needed to calculate the likelihood that SNP $j$ belongs to the distribution $b_{j} \sim N\left( {0,\sigma^{2} } \right)$ or $b_{j} = 0$, as shown below.

### The log-likelihood ratio of $b_{j} \sim N\left( {0,\sigma^{2} } \right)$ versus $b_{j} = 0$

In order to write the likelihood under the model without an effect at SNP $j$, $b_{j} = 0$, we write the (co)variance matrix of the records as $Var\left( {\mathbf{y}} \right) = {\mathbf{I}}\sigma_{e}^{2} + {\mathbf{G}}_{ - j} = {\mathbf{R}}\sigma_{e}^{2}$. From the multivariate normal density function, the log-likelihood of this model is:

$$ LogL\left( b_{j} = 0 \right) = - \frac{1}{2}\left[ N\log \left( \sigma_{e}^{2} \right) + Log\left( \left| {\mathbf{R}} \right| \right) + \left( \mathbf{y} - \mathbf{1}\upmu \right)^{\prime } \mathbf{R}^{ - 1} \left( \mathbf{y} - \mathbf{1}\upmu\right)/\sigma_{e}^{2}  \right]. $$

For the model with a non-zero SNP effect, i.e. $b_{j} \sim N\left( {0,\sigma^{2} } \right)$, the variance of the records is:$Var\; ({\mathbf{y}} )= {\mathbf{R}}\sigma_{e}^{2} + {\mathbf{x}}_{j} {\mathbf{x}}_{j}^{{\prime }} \sigma^{2} .\;{\text{The}}\;{\text{inverse}}\;{\text{of}}\;{\text{Var}}\left( {\mathbf{y}} \right)\;{\text{is}}:$

$$ \left[ {Var\left( {\mathbf{y}} \right)} \right]^{ - 1} = {\mathbf{R}}^{ - 1} /\sigma_{e}^{2} - {\mathbf{R}}^{ - 1} {\mathbf{x}}_{j} \left( {{\mathbf{x}}_{j}^{{\prime }} {\mathbf{R}}^{ - 1} {\mathbf{x}}_{j} /\sigma_{e}^{2} + 1/\sigma^{2} } \right)^{ - 1} {\mathbf{x}}_{j}^{{\prime }} {\mathbf{R}}^{ - 1} /\sigma_{e}^{4} = \left[ {{\mathbf{R}}^{ - 1} - {\mathbf{R}}^{ - 1} {\mathbf{x}}_{j} {\mathbf{x}}_{j}^{{\prime }} {\mathbf{R}}^{ - 1} /\left( {{\mathbf{x}}_{j}^{{\prime }} {\mathbf{R}}^{ - 1} {\mathbf{x}}_{j} + \lambda } \right)} \right]/\sigma_{e}^{2} . $$

And the determinant of $Var\left( {\mathbf{y}} \right)$ is:

$$ \left| {Var\left( {\mathbf{y}} \right)} \right| = \sigma_{e}^{2N} \left| {\mathbf{R}} \right|\sigma^{2} \left( {\frac{1}{{\sigma^{2} }} + {\mathbf{x}}_{j}^{{\prime }} {\mathbf{R}}^{ - 1} {\mathbf{x}}_{j} /\sigma_{e}^{2} } \right) = \sigma_{e}^{2N} \left| {\mathbf{R}} \right|\lambda^{ - 1} \left( {\lambda + {\mathbf{x}}_{j}^{{\prime }} {\mathbf{R}}^{ - 1} {\mathbf{x}}_{j} } \right). $$

The log-likelihood of a nonzero effect at SNP $j$ is most conveniently expressed as a deviation from the log-likelihood of the model when $b_{j} = 0$, i.e. as the loglikelihood ratio $LLR_{j} = LogL\left( {b_{j} \ne 0} \right) - LogL\left( {b_{j} = 0} \right)$:

$$ LLR_{j} = \frac{1}{2}\left[ {\log (\lambda ) - \log \left( {\lambda + {\mathbf{x}}_{j}^{{\prime }} {\mathbf{R}}^{ - 1} {\mathbf{x}}_{j} } \right) + \frac{{[{\mathbf{x}}_{j}^{{\prime }} {\mathbf{R}}^{ - 1} \left( {{\mathbf{y}} - {\mathbf{1}}\upmu} \right)]^{2} /\sigma_{e}^{2} }}{{{\mathbf{x}}_{j}^{{\prime }} {\mathbf{R}}^{ - 1} {\mathbf{x}}_{j} + \lambda }}} \right], $$

where the term (${\mathbf{x}}_{j}^{{\prime }} {\mathbf{R}}^{ - 1} {\mathbf{x}}_{j} + \lambda$) is obtained from Eq. (5) and the term $[{\mathbf{x}}_{j}^{{\prime }} {\mathbf{R}}^{ - 1} \left( {{\mathbf{y}} - 1\upmu} \right)]$ from Eq. (4) (see previous Section).

### Ratio of posterior probabilities and BayesC estimates of SNP effects

The *LLR* is combined with the log-prior-ratio, $Log(\uppi) - Log(1 -\uppi)$ into the log-posterior-probability-ratio:

$$ LPPR_{j} j = LLR_{j} + Log(\uppi) - Log(1 -\uppi). $$

The posterior probability of SNP $j$ having a nonzero effect is now:

$$ PP_{j} = \frac{1}{{1 + { \exp }\left( { - LPPR_{j} } \right)}}. $$

To approximate the BayesC analysis, we remain within the realm of linear models and translate the posterior probabilities into individual variances of SNP effects, $D_{j}$:

$$ D_{j} = {\text{PP}}_{j} \times \sigma^{2} . $$

The BayesC estimates of SNP effects, ${\mathbf{b}}_{c}$, can be obtained from a linear model with SNP weights proportional to $D_{j}$ by assuming $Var\left( {{\mathbf{b}}_{c} } \right) = {\mathbf{D}}\sigma_{b}^{2}$, where ${\mathbf{D}}$ is a diagonal matrix with elements $\tilde{D}_{j}$, with $\tilde{D}_{j} = D_{j} k/\left( {\mathop \sum \nolimits_{j} D_{j} } \right)$, i.e. $D_{j}$ is scaled such that the sum of the SNP variances (i.e. trace(${\mathbf{D}})\sigma_{b}^{2}$) is the same as in the SNP-BLUP analysis (i.e. trace(${\mathbf{D}})\sigma_{b}^{2} = k\sigma_{b}^{2}$).

In the BayesC analysis, the variance of the components ${\mathbf{s}}_{c}$ becomes:

$$ Var ({\mathbf{s}}_{c} )= Var\left( {{\mathbf{V}}^{{\prime }} {\mathbf{b}}_{c} } \right) = {\mathbf{V}}^{{\prime }} {\mathbf{DV}}\sigma_{b}^{2} . $$

And the BayesC estimates ${\hat{\mathbf{s}}}_{c}$ are obtained from Henderson’s MME:

6 $$ \left[ {\begin{array}{*{20}c} N & {\mathbf{0}} \\ {\mathbf{0}} & {{\mathbf{S}}^{2} + ({\mathbf{V}}^{{\prime }} {\mathbf{DV}})^{ - 1} \lambda_{b} } \\ \end{array} } \right]\left[ {\begin{array}{*{20}c} {\hat{\mu }} \\ {\widehat{{\varvec{s}_{c} }}} \\ \end{array} } \right] = \left[ {\begin{array}{*{20}c} {{\mathbf{1}}^{{\prime }} {\mathbf{y}}} \\ {{\mathbf{SU}}^{{\prime }} {\mathbf{y}}} \\ \end{array} } \right] . $$

Unfortunately, the coefficient matrix of these MME is no longer diagonal. The size of matrix ${\mathbf{V}}^{{\prime }} {\mathbf{DV}}$ is the number of components squared, thus, as long as the number of components is not too large (e.g. < 10,000), computation of its inverse is reasonably easy. From ${\hat{\mathbf{s}}}_{c}$, the SNP effects can be obtained as:

7 $$ {\hat{\mathbf{b}}}_{c} = Cov\left( {{\mathbf{b}}_{c} ,{\mathbf{s}}_{c}^{{\prime }} } \right)[Var ({\mathbf{s}}_{c} )]^{ - 1} {\hat{\mathbf{s}}}_{c} = {\mathbf{DV}}\left( {{\mathbf{V}}^{{\prime }} {\mathbf{DV}}} \right)^{ - 1} {\hat{\mathbf{s}}}_{c} . $$

A more formal derivation of Eq. (7) is provided in “Appendix”. The BayesC estimates of the breeding values of the animals are obtained as ${\mathbf{US}}\hat{\mathbf s}_{c}$. Note that the BayesC algorithm described above does not require iteration, which makes it computationally fast.

### Analysis of a simulated WGS data

WGS data were simulated to demonstrate the calculations and evaluate their results. The simulated species had 20 chromosomes of 1 Morgan (10^8^ bp) each. Simulation of WGS data followed the approach of [6], except that their scaling argument was not applied here, i.e. the computational costs were not scaled down. The historical effective population size was 1000, which also reflects its actual size since simulation of new generations followed Wright’s idealized population structure. In order to create LD and mutation-drift equilibrium, the historical population was simulated for 10,000 generations. In the last of the 10,000 generations, population size was increased to 10,000 individuals, which represented the reference population. The per meiosis and per base pair mutation rate was 10^−8^, and mutations followed the infinite sites model. This resulted in 531,836 SNPs with minor allele frequencies (MAF) higher than 0.01 in the reference population, in which SNP effects were estimated. Per chromosome, 200 SNPs with MAF higher than 0.01 were randomly sampled as causative SNPs, i.e. 4000 causative SNPs in total. Genotypes were standardized to the values of $- 2p_{j} /\surd \left( {2p_{j} \left( {1 - p_{j} } \right)} \right)$, $\left( {1 - 2p_{j} } \right)/\surd \left( {2p_{j} \left( {1 - p_{j} } \right)} \right)$, and $\left( {2 - 2p_{j} } \right)/\surd \left( {2p_{j} \left( {1 - p_{j} j} \right)} \right)$ for genotypes ‘0 0’, ‘0 1’ and ‘1 1’, respectively, where $p_{j}$ is the frequency of allele 1, and collected in the genotype matrix ${\mathbf{X}}$.

True genetic values of the animals were obtained as:

8 $$ {\mathbf{TBV}} =\upalpha{\mathbf{Xt}} , $$

where ${\mathbf{t}}$ is a ($531{,}836 \times 1$) vector of SNP effects, which were sampled from a normal distribution for the 4000 causative SNPs and were set to 0 for non-causative SNPs; and $\upalpha$ is a scaling factor which was chosen such that the variance of ${\mathbf{TBV}}$ in the reference population was 1. Phenotypes were obtained by adding random noise sampled from the distribution $N(0,1$) to ${\mathbf{TBV}}$, resulting in a heritability of 0.5. To estimate SNP effects, we used the phenotypes on the 10,000 animals in the reference population and their genotype matrix ${\mathbf{X}}$, and applied SNP-BLUP Eq. (2), our deterministic BayesC method Eq. (7), or a, MCMC based BayesC algorithm [15]. For the BayesC analyses, it was assumed that $\uppi = 0.01$ and that each causative SNP explained a proportion 0.001 of the total genetic variance. Heritabilities and genetic and environmental variances were assumed known for all analyses.

We assumed that the estimates of SNP effects were used in later generations to predict EBV, thus 10 more generations were simulated by applying Wright’s idealized population structure. The effective size in these 10 descending generations was reduced to 100 in order to increase genetic drift towards values that are realistic for livestock populations. In these generations, ${\mathbf{TBV}}$ were calculated using Eq. (8) and the correlation between ${\mathbf{TBV}}$ and estimates of the breeding values based on the estimated SNP effects was used as a measure of the accuracy of GS. The results were based on only four replicated simulations because the computational costs of these WGS data simulations and analyses were high, both in terms of computer time and disk space.

### SVD of the simulated WGS data

The ‘economy’ version of ${\text{SVD}}$ was conducted on the standardized genotypes matrices from each of the 20 chromosomes separately (average size $10{,}000 \times 26{,}592$), where ‘economy’ implies that components with singular values equal to zero were not computed. For all chromosomes, the 6000 largest singular values explained more than 95% of the total variance, i.e. $Trace\left( {{\mathbf{S}}_{6000}^{2} } \right) > 0.95*Trace\left( {{\mathbf{S}}^{2} } \right),$ where ${\mathbf{S}}_{6000}$ is the diagonal matrix with the 6000 largest singular values. Hence, the 6000 largest singular values were retained for each chromosome and, for each chromosome, we defined ${\mathbf{T}}_{6000} = {\mathbf{U}}_{6000} *{\mathbf{S}}_{6000}$. Next, an overall ${\text{SVD}}$ was performed for ${\mathbf{T}}_{120000} = \left[ {{\mathbf{T}}_{6000\left( 1 \right)}  {\mathbf{T}}_{6000\left( 2 \right)} \ldots {\mathbf{T}}_{{6000\left( {20} \right)}} } \right]$, where subscript ($i$) denotes chromosome $i$:

$$ {\text{SVD}}\left( {{\mathbf{T}}_{120000} } \right) = {\mathbf{U}}_{0} {\mathbf{S}}_{0} {\mathbf{V}}_{0}^{{\prime }} , $$

where ${\mathbf{T}}_{120000}$ has dimensions $10,000 \times 120,000$, and ${\mathbf{U}}_{0}$, ${\mathbf{S}}_{0}$ and ${\mathbf{V}}_{0}$ denote the ${\text{SVD}}$ of ${\mathbf{T}}_{120000}$. The overall ${\text{SVD}}$ of all genotypes can be obtained as:

$$ {\text{SVD}}\left( {\mathbf{X}} \right) = {\mathbf{U}}_{0} {\mathbf{S}}_{0} {\mathbf{V}}_{0}^{{\prime }} , $$

where

$$ {\mathbf{V}} = \left[ {\begin{array}{*{20}c} {{\mathbf{V}}_{6000\left( 1 \right)} {\mathbf{V}}_{{0\left( {{\text{rows }} \in {\text{chrom}}1} \right)}} } \\ \vdots \\ {{\mathbf{V}}_{{6000\left( {20} \right)}} {\mathbf{V}}_{{0\left( {{\text{rows }} \in {\text{chrom}}20} \right)}} } \\ \end{array} } \right], $$

with ${\mathbf{V}}_{6000\left( i \right)}$ denoting the ${\mathbf{V}}$ matrix from the ${\text{SVD}}$ of each chromosome $i$.

## Results

**Fig. 1:**
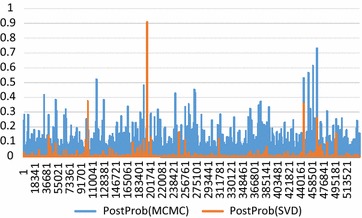
Manhattan plot of posterior probabilities across the genome

Figure 1 compares the posterior probabilities from the ${\text{SVD}}$-based BayesC analysis and the MCMC-based BayesC analysis. Because there are 4000 QTL, i.e. QTL are regularly distributed along the genome, the QTL positions are not indicated in Fig. 1. Generally, both analyses agreed on where the regions with the highest posterior probability are, but the ${\text{SVD}}$-based analysis showed much more pronounced QTL signals than the MCMC analysis. Thus, it appears that the assumption of the linear model involved in the ${\text{SVD}}$ analysis makes it overconfident about some SNP positions. The MCMC analysis implicitly accounts for the mixture distribution of the model, which results in an increase in SNPs with small estimates of effects and a decrease in SNPs with large estimates, which agrees with the results of [8]. Also, the ${\text{SVD}}$-based posterior probabilities generally seem smaller than those from the MCMC analysis. The sum of the posterior probabilities for the MCMC and ${\text{SVD}}$-based analyses were 3884 and 67, respectively (result not shown elsewhere). Thus, the sum of the posterior probabilities of the MCMC-based analyses was much closer to the actual number of QTL, i.e. 4000.

**Fig. 2:**
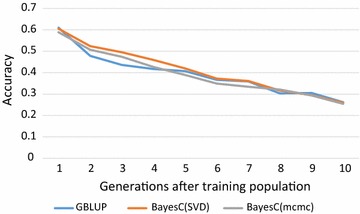
Accuracies of genomic selection over 10 descending generations, where SNP effects were estimated in generation 0 using whole-genome sequence data

The profile of the accuracy of GS using WGS data over 10 generations of descendants is in Fig. 2. All three analyses started with about the same accuracy and showed a decline of accuracies as the time between the reference and validation population increased. The accuracies of the SNP-BLUP analysis tended to drop somewhat more during intermediate generations 2 to 5 compared to those of the ${\text{SVD}}$-BayesC analysis. From generation 5 onwards, the accuracy of all analyses dropped at similar rates. The MCMC-BayesC analysis yielded similar accuracies as the ${\text{SVD}}$-based analysis, but during the intermediate generations its accuracy was between that of SNP-BLUP and ${\text{SVD}}$-BayesC. The latter agrees with Fig. 1, where the SNP solutions of MCMC-BayesC are less skewed and thus more like SNP-BLUP solutions. In any case, the ${\text{SVD}}$-BayesC analysis appeared to be at least as accurate as the MCMC-BayesC analysis.

**Fig. 3:**
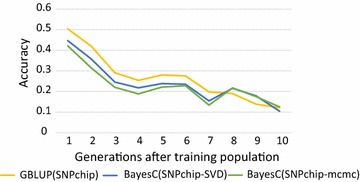
Accuracies of genomic selection using over 10 descending generations, where SNP effects were estimated in generation 0 using ~ 30 k SNP-chip data

Figure 3 shows the accuracy of GS using ~ 30 k SNP-chip data. In this situation, the SNP-BLUP analysis was more accurate, except from generation 8 onwards, for which accuracies were similar across methods. Both BayesC analyses had similar accuracies. When the causative mutations were not included in the genotype data, accurate GS appeared to depend on the prediction of the effects of SNP haplotypes that segregate in the population. The SNP-BLUP method appeared to achieve this better, probably because it uses all genotyped SNPs in the region to define haplotypes. Over generations, the original haplotypes are broken down by recombination and, thus, the accuracies of all methods decreased.

**Fig. 4:**
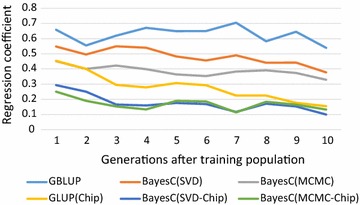
Regression coefficients of true on estimated breeding values over 10 descending generations where SNP effects were estimated in generation 0. Deviations of the regression coefficients from 1 measure the bias of the estimated breeding values

Figure 4 shows the estimates of regression coefficients of true on estimated breeding values over time. For unbiased prediction, these regression coefficients should be 1, but all methods showed some bias in the sense that the distribution of the estimated breeding values was too large (regression coefficients less than 1 shrink the GEBV). Although the SNP-BLUP method resulted in the smallest bias, it was also somewhat biased, possibly because the SNP effects were estimated in a dataset with few close relatives (due to the large effective size of the reference population). Thus, the genomic relationship matrix, ${\mathbf{G}}$, was very similar to the residual covariance matrix, apart from some structure due to small genetic relationships and, thus, the SNP estimates could easily pick-up some covariances due to the randomness of residuals. This effect will be enhanced for the Bayesian variable selection models, which specifically search for SNP genotypes that correlate well with the phenotypes and thus also with residuals. In the case of the SNP-chip data, these biases were even larger due to imperfect LD between the SNPs and the QTL.

### Computing times

Table 1 shows the computing times for the alternative GS models. The MCMC BayesC method required almost 2 days and, thus, is impractical, especially when the size of datasets exceeds 10,000 animals and half a million SNPs. ${\text{SVD}}$ was performed by the Lapack library routines (http://www.netlib.org/lapack/), which provides parallel algorithms for this task (10 parallel processors were used here). The ${\text{SVD}}$ was the most time-demanding step in ${\text{SVD}}$-BayesC, with 8.5 min per chromosome (note that this could be performed for all chromosomes in parallel) and 25 min for the overall ${\text{SVD}}$. The computing time of the ${\text{SVD}}$ of a matrix with dimension ($n \times m$) increases proportionally to $m*n^{2}$, where $n$ is the smaller dimension (usually the number of animals) and $m$ the larger dimension (usually the number of SNPs or the number of components involved in the overall ${\text{SVD}}$). Thus, at a constant number of animals, the chromosome-wise ${\text{SVD}}$ would increase only linearly with the number of SNPs. Since the required number of components per chromosome is expected to increase only marginally as the number of SNPs increases, computation time of the overall ${\text{SVD}}$ should increase only marginally. Computing times for the overall ${\text{SVD}}$ are expected to increase linearly with the number of chromosomes (assuming the chromosomes are of equal size and the number of components involved in the overall ${\text{SVD}}$ exceeds the number of animals). The memory requirements of the ${\text{SVD}}$ are $8\left[ {n^{2} + \left( {m + 1} \right)n} \right]$ bytes, assuming double precision calculations, and thus increase in a quadratic manner with the smallest dimension and linearly with the largest dimension of the genotype matrix. Because of the large storage requirements of the results of the ${\text{SVD}}$ of a large matrix, it may be beneficial to store ${\text{SVD}}$ results in a compressed form, although we did not attempt this here.

**Table 1:** Computing times with a dual core Xeon(R) CPU E5-2620 v2 (2.10 GHz) machine with 24 processors

Task	Number of times required^a^	Wall-time			Number of processors used
		Days	Hours	Minutes	
MCMC_BayesC	1	1	19	28	6
SVD (one chromosome)	20			8.5	10
SVD (overall)	1			25.1	10
SVD-based BayesC^b^	1			2.2	1
SVD-based SNPBLUP^b^	1			1.5	1

^a^Tasks that can be performed in parallel on different nodes; 20 reflects the number of chromosomes

^b^ ${\text{SVD}}$ has been performed before hand

## Discussion

An ${\text{SVD}}$-based variable selection model was developed that is computationally fast, assuming that the ${\text{SVD}}$ of the genotype matrix ${\mathbf{X}}$ was performed previously. It was shown that ${\text{SVD}}$ of ${\mathbf{X}}$ facilitates the calculation of the PEV of SNP effects, which were used in a BayesC setting to calculate posterior probabilities of a QTL. Although the posterior probabilities were generally lower than those from a MCMC BayesC analysis, the accuracies of prediction were competitive compared to those of an MCMC analysis. The persistency of the accuracies across generations of descendants was tested in a worse-case scenario, i.e. without updating of the reference population. The persistency of accuracy over generations was similar for the ${\text{SVD}}$- and MCMC-based predictions and slightly higher than for the SNP-BLUP analysis, at least during intermediate generations when using WGS data.

When 30 K SNP-chip data were used, the MCMC and ${\text{SVD}}$-BayesC analyses also yielded similar results, but their accuracy of prediction was slightly lower than that of SNP-BLUP. Apparently, the number of SNPs with effects is so large that assuming a priori that all SNPs have effects, as in the SNP-BLUP model, is beneficial, a situation that was also described by Daetwyler et al. [16].

The difference between the posterior probabilities, $PP_{j}$, from the ${\text{SVD}}$- and the MCMC-based analyses may be due to the fact that the former assumes that the variance at locus $j$ is known without error (resulting in Eq. (3) to predict the PEV of the SNP effect), whereas the MCMC analysis accounts for the uncertainty of the variance at locus $j$. This appears to result in stronger $PP_{j}$ signals for the ${\text{SVD}}$-based compared to the MCMC-based analysis in some chromosomal regions. When this is undesirable, higher values of $\uppi$ can be used to spread the genetic effects over more SNPs. Although 4000 QTL were randomly scattered across the genome, the regions with large $PP_{j}$ signals may have a high density of QTL. Nevertheless, since the MCMC analysis may be seen as the ‘golden standard’, we consider the stronger QTL signals from ${\text{SVD}}$-based posterior probabilities to be anti-conservative.

We assumed that the value of $\uppi$ was known and equal to the number of QTL divided by the number of SNPs. This assumes that effectively only one SNP is needed to predict a QTL (although a number of SNPs with reduced $PP_{j}$ might actually pick up the QTL effect), which has been found to result in similar accuracies as using an optimized value of $\uppi$ [17]. However, in real data, the number of QTL is unknown but the optimal value of $\uppi$ can be found by cross-validation [18]. ${\text{SVD}}$-BayesC is well suited for such cross-validation computations, because of its computational speed and because the ${\text{SVD}}$ of the genotype matrix needs to be performed only once. Note that the choice of $\uppi$ defines the variance of SNP effects $\sigma^{2}$, based on equation $N_{SNPs}\uppi\sigma^{2} = \sigma_{g}^{2}$, where $N_{SNPs}\uppi\sigma^{2}$ equals the total genetic variance assumed by the BayesC model, and $\sigma_{g}^{2}$ the (assumed) genetic variance of the trait.

The computational speed of the ${\text{SVD}}$-based BayesC analysis depended heavily on the computation of the ${\text{SVD}}$. We performed the ${\text{SVD}}$ separately for each chromosome, but different situations may call for different strategies to perform the ${\text{SVD}}$. For instance, ${\text{SVD}}\left( {\mathbf{X}} \right)$ may have been already obtained for other reasons than the BayesC analysis, e.g. to perform large-scale national evaluations by predicting only the components in order to reduce computations (Ødegård, Indahl, Stranden, Meuwissen: Large-scale genomic prediction using singular value decomposition of the genotype matrix; Accepted in GSE). In the case of a real WGS analysis, the number of SNPs is often substantially larger than in our simulation, e.g. due to a very large effective population size in the distant past, which generated many SNPs that are still segregating. If the SVD of a chromosome is too large, it can be performed per chromosome segment instead of per chromosome, which is a straightforward extension of the ${\text{SVD}}$ analysis by chromosome adopted here. In a subsequent study, we intend to perform the ${\text{SVD}}$ on real WGS data.

In situations where the family structure is not strong (as was the case in our simulated data), the per chromosome components are approximately unrelated and the final ${\text{SVD}}$ on the combined components (${\text{SVD}}\left( {{\mathbf{T}}_{120000} } \right)$) can be omitted. In this case, ${\text{SVD}}\left( {\mathbf{X}} \right) = {\mathbf{USV}}^\prime$ , with ${\mathbf{U}} = \left[ {{\mathbf{U}}_{\left( 1 \right)}  {\mathbf{U}}_{\left( 2 \right)} \ldots {\mathbf{U}}_{\left( l \right)} } \right]$, ${\mathbf{S}} = {\text{diag}}\left( {{\mathbf{S}}_{\left( i \right)} } \right)$, and ${\mathbf{V}} = {\text{diag}}\left( {{\mathbf{V}}_{\left( i \right)} } \right)$, where subscript ($i$) denotes matrices from the per chromosome ${\text{SVD}}$ of chromosome $i$; and ${\text{diag}}\left( {{\mathbf{V}}_{\left( i \right)} } \right)$ denotes a block-diagonal matrix with the diagonal blocks containing the ${\mathbf{V}}_{\left( i \right)}$ matrices. Since this ${\text{SVD}}$ results only in approximately independent components, Eq. (3) holds only approximately. Whether this approximation is sufficiently accurate can be investigated by checking whether ${\mathbf{U}}^{{\prime }} {\mathbf{U}} \approx {\mathbf{I}}$, and will depend on the family structure of the population. In an ultimate test, one can use Eq. (3) (knowing that it holds only approximately) and check the accuracy of the resulting BayesC analysis by cross-validation. In this analysis, Eqs. (6) and (7) can be used to estimate the SNP-BLUP marker effects [instead of Eq. (1)] in order to account for covariances between the components.

## Conclusions

After performing the ${\text{SVD}}$, the BayesC analysis developed here is computationally fast and comparable to SNP-BLUP calculations, whereas its accuracy is competitive compared to that of MCMC-based BayesC analyses. It may also be noted that the ${\text{SVD}}$ needs to be performed only once across all traits. Thus, when many traits need to be analysed, the computational costs of calculating ${\text{SVD}}\left( {\mathbf{X}} \right)$ are relatively small on a per trait basis. The profiles of the accuracies over generations showed that BayesC accuracies were slightly more persistent over an intermediate number of generations/meioses (2–5 generations) than the SNP-BLUP (or, equivalently, GBLUP) accuracies, which enables genomic predictions over longer genetic distances.

## Appendix

### SVD-based SNP-BLUP prediction with weighted SNP effects

The weighted SNP-BLUP model is:

$$ {\mathbf{y}} = {\mathbf{Xb}} + {\mathbf{e}} = {\mathbf{USV}}^{{\prime }} {\mathbf{b}} + {\mathbf{e}}, $$

where ${\mathbf{y}}$ is the vector of phenotypes; ${\mathbf{X}}$ is the matrix of standardised genotypes and ${\mathbf{USV}}^{{\prime }}$ represents the ${\text{SVD}}$ of ${\mathbf{X}}$; ${\mathbf{b}}$ is the vector of SNP effects with unequal variances, i.e. $Var ({\mathbf{b}} )= {\mathbf{D}}\sigma^{2}$ with ${\mathbf{D}}$ often being a diagonal matrix (representing the weights of the SNPs); and ${\mathbf{e}}$ is the vector of residuals with $Var ({\mathbf{e}} )= {\mathbf{I}}\sigma_{e}^{2}$. In the derivations below, ${\mathbf{D}}$ needs to be invertable but may contain off-diagonal elements.

The mixed model equations are:

$$ \left( {{\mathbf{X^{\prime}X}} + {\mathbf{D}}^{ - 1}\uplambda} \right){\hat{\mathbf{b}}} = {\mathbf{X}}^{{\prime }} {\mathbf{y}}. $$

Using the ${\text{SVD}}$ of ${\mathbf{X}} = {\mathbf{USV}}^{{\prime }}$ and using ${\mathbf{U}}^{{\prime }} {\mathbf{U}} = {\mathbf{I}}$:

9 $$ \left( {{\mathbf{VS}}^{2} {\mathbf{V}}^{{\prime }} + {\mathbf{D}}^{ - 1}\uplambda} \right){\hat{\mathbf{b}}} = {\mathbf{VSU}}^{{\prime }} {\mathbf{y}} . $$

Alternatively, the components ${\mathbf{s}} = {\mathbf{V}}^{{\prime }} {\mathbf{b}}$ are estimated directly using the model:

$$ {\mathbf{y}} = {\mathbf{USV}}^{{\prime }} {\mathbf{b}} + {\mathbf{e}} = {\mathbf{USs}} + {\mathbf{e}}, $$

with the (co)variance matrix of the components: $Var ({\mathbf{s}} )= {\mathbf{V}}^{{\prime }} Var ({\mathbf{b}} ){\mathbf{V}} = {\mathbf{V}}^{{\prime }} {\mathbf{DV}}$.

The mixed model equations for the estimation of the components are:

10 $$ \left( {{\mathbf{S}}^{2} + ({\mathbf{V}}^{{\prime }} {\mathbf{DV}})^{ - 1}\uplambda} \right){\hat{\mathbf{s}}} = {\mathbf{SU}}^{{\prime }} {\mathbf{y}} , $$

which are the equations used in the main text.

Rewritting Eq. (10) towards the estimation of SNP effects yields:

$$ ({\mathbf{S}}^{2} {\mathbf{V}}^{{\prime }} {\mathbf{DV}} + {\mathbf{I}}\uplambda)({\mathbf{V}}^{{\prime }} {\mathbf{DV}})^{ - 1} {\hat{\mathbf{s}}} = {\mathbf{SU}}^{{\prime }} {\mathbf{y}} $$

$$ ({\mathbf{DVS}}^{2} {\mathbf{V}}^{{\prime }} {\mathbf{DV}} + {\mathbf{DV}}\uplambda)({\mathbf{V}}^{{\prime }} {\mathbf{DV}})^{ - 1} {\hat{\mathbf{s}}} = {\mathbf{DVSU}}^{{\prime }} {\mathbf{y}} $$

$$ ({\mathbf{VS}}^{2} {\mathbf{V}}^{{\prime }} + {\mathbf{I}}\uplambda)[{\mathbf{DV}}({\mathbf{V}}^{{\prime }} {\mathbf{DV}})^{ - 1} {\hat{\mathbf{s}}}] = {\mathbf{VSU}}^{{\prime }} {\mathbf{y}} $$

which are the same equations as (9), except that the solution is written in a different form. Since the same equations have the same solutions (assuming there is a unique solution), estimates of the SNP effects can be obtained from the estimates of the components ${\hat{\mathbf{s}}}$ as:

$$ {\hat{\mathbf{b}}} = {\mathbf{DV}}\left( {{\mathbf{V^{\prime}DV}}} \right)^{ - 1} {\hat{\mathbf{s}}}. $$
